# Cigarette purchasing behaviors and financial burden of cigarette spending after a major tobacco tax increase in California: A descriptive analysis of household panel data

**DOI:** 10.1016/j.pmedr.2025.103352

**Published:** 2025-12-22

**Authors:** Dian Gu, Hai-Yen Sung, Tingting Yao, Yingning Wang, Courtney Keeler, Wendy Max

**Affiliations:** a Institute for Health & Aging, School of Nursing, University of California San Francisco, San Francisco, CA 94143, United States; b The Center for Tobacco Control Research and Education, University of California San Francisco, San Francisco, CA 94143, United States; c Department of Population Health Sciences, School of Nursing, University of San Francisco, San Francisco, CA 94143, United States

**Keywords:** Tobacco tax policy, Cigarette purchase, Cigarette spending, Financial burden, Tobacco disparity

## Abstract

**Objective:**

To examine the longitudinal patterns in cigarette purchasing behaviors and financial burden of cigarette spending among California households following the implementation of Proposition 56, which increased cigarette excise taxes by $2-per-pack in April 2017.

**Methods:**

Using longitudinal NielsenIQ Consumer Panel data, we identified a cohort of 2324 California households that participated continuously from 2016 through 2022. We compared outcomes — cigarette purchasing behaviors and financial burden of cigarette spending — from the 2016 baseline to each follow-up year and the 5-year average during 2018–2022 (post-implementation), both overall and by income group.

**Results:**

In 2016, 10.6 % of California households purchased cigarettes; this percentage declined steadily to 4.2 % in 2022 (*p* < 0.01). The reduction over five years was significantly smaller among low-income households compared to middle-income (2.1 % vs. 5.1 %, *p* < 0.01) and high-income groups (2.1 % vs. 3.8 %, *p* < 0.01). Among low-income households that continued purchasing cigarettes, annual cigarette spending increased from $651 at baseline to $1053 in 2021 (*p* = 0.04) and $1343 in 2022 (*p* < 0.01).

**Conclusions:**

The percentage of California households purchasing cigarettes declined across all income groups over five years after Proposition 56, with the smallest declines among low-income households. In this group, annual cigarette spending increased significantly in the fourth and fifth post-implementation years.

## 1 Introduction

Increasing tobacco taxes is considered an effective strategy for reducing cigarette smoking (Chaloupka et al., 2012). Every 10 % increase in the price of cigarettes leads to a 2–6 % reduction in overall cigarette demand among adults, an effect attributed to both decreased smoking prevalence and decreased cigarette consumption among smokers (Chaloupka and Warner, 2000; Gallet and List, 2003; IARC, 2011). However, these gains are not shared equally. One concern is whether cigarette taxes are equitable; i.e., whether the percentage of income paid for the tax is the same across income groups (Chaloupka and Warner, 2000). Some economists argue that while cigarette taxes are regressive because low-income people smoke more and spend a greater percentage of income paying the tax than do higher-income people, increasing cigarette taxes may reduce the degree of regressivity of cigarette taxes if it leads to a greater reduction in smoking for low-income versus higher-income smokers (Bader et al., 2011; Chaloupka and Warner, 2000; Warner, 2000).

Limited empirical research has estimated how tobacco tax increases are related to smokers' financial burden. Using cross-sectional survey data, one study found that between 2003 and 2004 and 2010–2011 as New York's state cigarette tax increased from $1.50/pack to $4.35/pack, the percentage of income spent on cigarettes increased significantly among lowest-income smokers but not among highest-income smokers (Farrelly et al., 2012). Given the crowding-out effect of tobacco spending (Sánchez and Gómez, 2024; Swarnata et al., 2024), a greater burden of cigarette spending suggests that smokers may forgo basic necessities, which would negatively affect the well-being of household members (Sánchez and Gómez, 2024; Swarnata et al., 2024). Therefore, examining how tobacco tax increases are associated with smokers' financial burden, especially for low-income smokers, is crucial in the evaluation of tobacco taxation policy.

On April 1, 2017, California implemented Proposition 56 (Prop 56), a voter-approved initiative to raise the state's cigarette excise tax by $2 per pack (Keeler et al., 2020). This study examines the longitudinal patterns of cigarette purchasing behaviors and financial burden of cigarette spending for five years after Prop 56 by following a cohort of California households from 2016 (baseline) through 2022 and assesses whether patterns differed by income levels.

## 2 Methods

### 2.1 Study design and population

We conducted a longitudinal study by analyzing 2016–2022 NielsenIQ Consumer Panel (NCP) data, which contains information on weekly purchases of consumer products from a nationally representative panel of households in the United States. The NCP data also contains detailed demographic and geographic household information and product characteristics. The study cohort comprised 2324 California households that participated in the Panel continuously from 2016 to 2022. We classified households into three income groups for each year based on how self-reported household incomes compared to federal poverty level (FPL) thresholds (Census Bureau, 2025) for that year and household size: low-income (<200 % FPL), middle-income (200–399 % FPL), and high-income (≥400 % FPL). The original categorical income variable was converted to a continuous measure using the midpoint of each category. For the top income category (≥$100,000), the midpoint was calculated as twice the lower boundary (Pasta, 2009).

### 2.2 Measures

We examined two cigarette purchasing behavior variables: 1) whether a household purchased any cigarettes in a given year, and 2) annual number of total packs (20-cigarettes) purchased by households that purchased any cigarettes in a given year (hereafter referred to as cigarette-purchasing households). We examined two financial burden of cigarette spending variables: 1) annual spending on cigarettes, inflated to 2022 US dollars (U.S. Bureau of Labor Statistics, 2025), and 2) percentage of annual household income spent on cigarettes. Financial burden variables were analyzed only among cigarette-purchasing households.

### 2.3 Statistical analysis

We conducted a descriptive analysis to examine the annual patterns of outcomes from 2016 (baseline) to 2022. Because Prop 56 took effect in April 2017, we omitted 2017 from the analysis. For all households and each income group, we calculated the mean of each outcome variable for 2016 and each follow-up year (2018–2022). We also calculated the 5-year average of each outcome in 2018–2022 and the absolute change between 2016 and the 5-year average.

We assessed whether there was a statistically significant change in the outcome between 2016 and each follow-up year using a household-level generalized estimating equations (GEE) model. In the model, the dependent variable was the outcome measure, and the key independent variables included five yearly indicators (2018 vs. 2016, 2019 vs. 2016, etc.). The model controlled for covariates: household composition, presence of children, type of residence, race/ethnicity, household head's age, education, employment status, marital status, and urban/rural status. The GEE model was estimated separately for all households combined and each income group. The *p*-value for the yearly indicator was used to determine the assessment. We also assessed whether the change in the outcome between 2016 and each follow-up year was significantly different across income groups by including all households in an expanded GEE model, which added the income group indicators and the interaction terms between income group indicators and yearly indicators. The *p* value for the estimated interaction term was used to determine the assessment.

Similarly, we assessed whether there was a statistically significant change in the outcome between 2016 and the 5-year average and whether such change was significantly different across income groups by estimating the above-mentioned GEE models except that we replaced the five yearly indicators with a binary indicator for Prop 56 (=0 for 2016; =1 for other years).

To assess the impact of attrition on statistical significance and the robustness of our results, we conducted a sensitivity analysis comparing the outcomes between 2016 and 2018 using 3883 households that participated in both 2016 and 2018.

All analyses were performed using SAS V.9.4 procedures that accounted for NCP sampling weights. A two-tailed *p* < 0.05 was considered statistically significant.

The study was deemed exempt from review by the Institutional Review Board at the University of California, San Francisco.

## 3 Results

Table 1 shows the distribution of the study cohort in 2016. Table 2 shows that 10.6 % of California households purchased cigarettes in 2016. After Prop 56, this percentage declined to 8.3 % in 2018 (*p = 0.01*) and continued to decline significantly over time to 4.2 % in 2022 (*p < 0*.01), averaging 6.7 % during the 5-year follow-up with an absolute change of −3.9 % (*p* < 0.01) from 2016. By income groups, after Prop 56, a significant declining trend was observed for middle- and high-income groups with an absolute change of −5.1 % (*p* = 0.01) and − 3.8 % (*p* < 0.01), respectively. For the low-income group, the cigarette-purchasing percentage did not decrease until 2019, but the decrease was not statistically significant until 2022; the absolute change between 2016 and the 5-year average was −2.1 % (*p = 0.04*), which was significantly smaller than that for middle- *(p* < 0.01*)* and high-income households (*p* < 0.01*)*.

**Table 1:** Sample Distribution by Household Characteristics among the Study Cohort of 2324 California Households that participated in the NielsenIQ Consumer Panel in every year from 2016 through 2022, California, United States, at baseline (2016).

Covariates	N (%)
*Income level*	
Low-income	371 (26.2)
Middle-income	644 (22.0)
High-income	1309 (51.8)
*Household Composition*	
Single female	396 (16.6)
Single male	277 (10.8)
Multiple adults	1651 (72.6)
*Presence of children aged < 18 years*	
No	1941 (75.0)
Yes	383 (25.0)
*Type of residence*	
One family house	1813 (75.4)
Two+ family house/Mobile home or trailer	511 (24.6)
*Race/ethnicity*	
Non-Hispanic White	1354 (50.0)
Hispanic	331 (25.9)
Non-Hispanic Black	211 (6.2)
Non-Hispanic Other	428 (17.9)
*Age of household head*	
Early- to mid-life adults (<55 years)	814 (46.8)
Later-life adults (≥55 years)	1510 (53.2)
*Education status of household head*	
≤ High School	266 (21.2)
Some college	629 (33.1)
≥ College	1429 (45.7)
*Employment status of household head*	
Not employed	298 (8.6)
Employed	2026 (91.4)
*Marital status of household head*	
Married	1410 (51.5)
Not married	914 (48.5)
*Urban/rural status*	
Rural	71 (2.6)
Urban	2253 (97.4)

Note: N = unweighted sample size. %: weighted column percentage; Low-income: <200 % FPL; Middle-income: 200 % to <400 % FPL; High income: ≥400 % FPL.

**Table 2:** Cigarette purchasing behaviors and financial burden of cigarette spending at baseline (2016) and during a 5-year follow-up period (2018–2022) among 2324 California households participating in the NielsenIQ Consumer Panel in every year from 2016 through 2022, California, United States.

	Baseline	Follow-up years						Comparison of the 5-year follow-up period vs. 2016		p-value of the yearly indicators in GEE model ⁎⁎				
	2016	2018	2019	2020	2021	2022	5-year average	Absolute change between 2016 and the 5-year average	p-value of the Prop 56 indicator in GEE model ⁎	2018 vs. 2016	2019 vs. 2016	2020 vs. 2016	2021 vs. 2016	2022 vs. 2016
Cigarette purchasing behavior outcomes:														
(1) % of HHs that purchased cigarettes														
All	10.6	8.3	8.2	7.0	6.0	4.2	6.7	−3.9	**<0.01**	**0.01**	**0.02**	**<0.01**	**<0.01**	**<0.01**
Low-income	12.9	13.9	12.4	9.9	11.0	6.9	10.8	−2.1	**0.04**^a^^,^^b^	0.89 c	0.63	0.16	0.30	**0.01**
Middle-income	11.3	7.6	7.7	7.6	5.6	2.4	6.2	−5.1	**0.01**^a^	**0.04**	**0.03**	**0.03**	**0.01**	**<0.01**
High-income	9.2	5.9	6.7	6.0	4.6	3.7	5.4	−3.8	**<0.01**^b^	**<0.01**^c^	**0.04**	**<0.01**	**<0.01**	**<0.01**
(2) No. of packs purchased by cigarette-purchasing HHs														
All	90.8	56.7	52.6	57.6	83.7	86.7	67.5	−23.3	0.07	**0.01**	**0.02**	0.27	0.66	0.81
Low-income	103.1	68.2	78.0	70.3	119.5	147.5	96.7	−6.4	0.17	0.12	0.77	0.45	0.22	0.40
Middle-income	121.8	34.6	51.3	36.8	76.1	89.6	57.7	−64.1	0.10	**0.04**	0.97	0.49	0.46	0.34
High-income	65.7	53.4	34.5	59.0	61.0	48.8	51.3	−14.4	0.06	0.14	**0.02**	0.43	0.55	0.10
Financial burden of cigarette spending outcomes:														
(1) Annual cigarette spending (inflated to 2022 US$) among cigarette-purchasing HHs														
All	$566	$483	$460	$511	$764	$765	$597	$31	0.60	0.45	0.40	0.86	0.08	0.14
Low-income	$651	$552	$661	$574	$1053	$1343	$837	$186	0.38	0.65	0.73	0.59	**0.04**	**<0.01**
Middle-income	$718	$310	$466	$345	$681	$806	$522	-$196	0.82	0.88	0.39	0.65	0.31	0.23
High-income	$424	$476	$310	$542	$588	$401	$463	$39	0.50	0.57	0.94	**0.04**	0.07	0.49
(2) % of annual HH income spent on cigarettes among cigarette-purchasing HHs														
All	1.7	1.3	1.7	1.2	2.0	2.4	1.7	0.0	0.86	0.36	0.67	0.64	0.22	0.26
Low-income	3.9	2.5	4.2	3.4	4.7	6.2	4.2	0.3	0.98	0.18	0.84	0.61	0.42	0.17
Middle-income	1.4	0.5	0.8	0.6	1.2	1.7	1.0	−0.4	0.52	0.09	0.89	0.31	0.76	0.97
High-income	0.2	0.3	0.2	0.3	0.3	0.2	0.3	0.1	0.46	0.37	0.31	0.15	0.39	0.62

Note: HH = household. Low-income: <200 % FPL; Middle-income: 200 % to <400 % FPL; High income: ≥400 % FPL.

⁎ The GEE model on the outcome was specified as a function of a binary post-policy indicator (=0 for 2016; =1 for 2018–2022) as well as covariates including household composition, presence of children, type of residence, race/ethnicity, household head's age, education, employment status, marital status, and urban/rural status.

⁎⁎ The GEE model on the outcome was specified as a function of five binary yearly indicator variables (reference year = 2016) and all the covariates.

a p-value <0.01 to test whether the change between 2016 and the 5-year average differs statistically significant for low- vs. middle-income group.

b *p*-value <0.01 to test whether the change between 2016 and the 5-year average differs statistically significantly for low- vs. high-income group.

c *p*-value =0.04 to test whether the change between 2016 and 2018 differs statistically significantly for low- vs. high-income group.

Table 2 also shows that among cigarette-purchasing households, the annual quantity of cigarettes purchased decreased immediately from 90.8 packs in 2016 to 56.7 packs in 2018 (*p* = 0.01) and 52.6 packs in 2019 (*p* = 0.02), then increased to 86.7 packs in 2022 (*p* = 0.81). Although per-pack prices increased immediately after Prop 56 (Supplemental Table S1), annual per-household cigarette spending initially decreased, then increased later. However, none of these annual spending estimates in the follow-up years were significantly different from 2016.

Stratified by income group, the annual quantity of cigarettes purchased by the low-income group decreased from 103.1 packs in 2016 to 68.2 packs in 2018, but reversed to a higher-than-baseline level in 2021 (119.5 packs) and 2022 (147.5 packs), though not statistically significant. For the middle-income group, quantity dropped significantly from 121.8 packs in 2016 to 34.6 packs in 2018 (*p* = 0.04) and rebounded to 89.6 packs in 2022 (*p* = 0.34). The high-income group had a significant decline from 65.7 packs in 2016 to 34.5 in 2019 (*p* = 0.02), followed by fluctuations. For each income group, the patterns of annual per-household cigarette spending during the 5-year follow-up generally mimicked the patterns of annual quantity of cigarette packs. Notably, among low-income cigarette-purchasing households, the annual cigarette spending increased significantly from $651 in 2016 to $1053 in 2021 (*p* = 0.04) and $1343 in 2022 (*p* < 0.01).

In 2016, low-income cigarette-purchasing households spent 3.9 % of household income on cigarettes compared to 1.4 % and 0.2 % among middle- and high-income households, respectively. In 2022, this percentage increased to 6.2 % among low-income households (*p* = 0.17), though the change was not statistically significant. In contrast, the percentage remained relatively stable among middle- and high-income households.

The results from the sensitivity analysis show that the estimated percentages of households purchasing cigarettes and percentages of income spent on cigarettes for each income group were almost the same as those from the main analysis but the *p*-values for 2016 vs. 2018 were lower (Supplemental Table S2). For the other two outcome variables, the magnitudes of the estimates differed slightly from the main analysis, but the direction of changes from 2016 to 2018 were consistent and p-values were generally lower.

## 4 Discussion

This is the first longitudinal study in the U.S. to analyze the patterns of cigarette purchasing behaviors and financial burden of cigarette spending over five years after a major cigarette tax increase. After Prop 56, the percentage of middle- and high-income households purchasing cigarettes decreased every subsequent year. There was not a significant decrease for low-income households until the fifth year and the 5-year average reduction was significantly smaller than that for middle- and high-income households. We also found that among households that continued to purchase cigarettes, all income groups reduced their quantity of cigarettes purchased in the first year but only the high-income group continued to cut down in later years. The low-income group even bought more cigarettes in the fourth and fifth years compared to baseline, although this increase was not statistically significant. These findings may challenge a conventional view that the poor are more price-responsive than the wealthy in reducing smoking prevalence and smoking intensity (Colman and Remler, 2008; Farrelly et al., 2001; Gruber and Kőszegi, 2004).

This study showed that among low-income households that continued to purchase cigarettes, cigarette spending increased significantly in 2021 and 2022. This could lead to crowding out of expenditures on life necessities (Sánchez and Gómez, 2024; Swarnata et al., 2024). This result also supports the horizontal equity (fairness within a given income group) argument by Remler (Remler, 2004) that cigarette taxes heavily burden low-income smokers who do not quit. Therefore, targeted cessation programs for low-income smokers may help mitigate the regressive financial burden of cigarette taxes.

We estimated that cigarette spending accounted for 3.9 % of household income among low-income cigarette-purchasing households in 2016, increasing to 6.2 % in 2022. These estimates are roughly consistent with the findings by Busch and colleagues (Busch et al., 2004) that average expenditures on tobacco products were 5.1 % of all consumer expenditures among low-income (<200 % FPL) smoking households in the U.S. in 1995–2001 and by Colman and Remler (Colman and Remler, 2008) that U.S. smokers in the lowest income tercile spent 7.7 % of income on cigarette purchases.

This study has limitations. First, our study excluded households not continuously participating in the longitudinal panel over 2016–2022; this attrition resulted in a reduced sample size. Our sensitivity analysis (*n* = 3883) showed that the 2016 vs. 2018 results were consistent with those from the main analysis (*n* = 2324) except with lower *p*-values, suggesting that some statistically non-significant findings from the main analysis may be due to decreased statistical power from attrition. Second, NielsenIQ data only captures scanner-based transactions, which might cause under-recorded cigarette purchases. Third, as a descriptive analysis, this study cannot provide causal inference. The post-implementation period overlapped with COVID-19 and legalized recreational cannabis sales in California starting January 2018. Besides, there might be unobservable factors that affected secular trends in household cigarette purchasing for California and other states. Without considering such factors, the observed changes after Prop 56 cannot be causally attributed to Prop 56. Nonetheless, given the lack of longitudinal research examining the long-term association between tobacco outcomes and tobacco tax increases, this study provides a first glimpse. Future longitudinal studies utilizing rigorous causal models are needed.

## 5 Conclusions

Over a 5-year period after Prop 56, our results indicate that the percentage of California households purchasing cigarettes declined across all income groups. However, declines were smallest among low-income households. Additionally, among low-income households that continued purchasing cigarettes, cigarette spending increased in the fourth and fifth years. Future research is needed to determine whether these changes were caused by Prop 56.

## CRediT authorship contribution statement

**Dian Gu:** Writing – review & editing, Writing – original draft, Software, Methodology, Formal analysis, Data curation, Conceptualization, Investigation, Validation. **Hai-Yen Sung:** Writing – review & editing, Writing – original draft, Supervision, Methodology, Funding acquisition, Conceptualization, Investigation, Project administration, Resources, Validation. **Tingting Yao:** Writing – review & editing, Investigation, Methodology, Resources, Validation. **Yingning Wang:** Writing – review & editing, Investigation, Validation. **Courtney Keeler:** Writing – review & editing, Investigation, Validation. **Wendy Max:** Writing – review & editing, Supervision, Investigation, Resources, Validation.

## Disclaimer

Researcher(s)’ own analyses calculated (or derived) based in part on data from Nielsen Consumer LLC and marketing databases provided through the NielsenIQ Datasets at the Kilts Center for Marketing Data Center at The University of Chicago Booth School of Business. The conclusions drawn from the NielsenIQ data are those of the researcher(s) and do not reflect the views of NielsenIQ. NielsenIQ is not responsible for, had no role in, and was not involved in analyzing and preparing the results reported herein.

## Funding

The study was supported by the California 10.13039/100005188Tobacco-Related Disease Research Program (TRDRP) Grant #28IR-0041.

## Declaration of competing interest

The authors declare that they have no known competing financial interests or personal relationships that could have appeared to influence the work reported in this paper.

## Appendix A Supplementary data

Supplementary material. Image 1
