# Supplementary material for: Cigarette purchasing behaviors and financial burden of cigarette spending after a major tobacco tax increase in California: A descriptive analysis of household panel data
Source: Prev Med Rep. 2025 Dec 22;61:103352. doi: 10.1016/j.pmedr.2025.103352 (PMC12808600; doi:10.1016/j.pmedr.2025.103352)
Supplement: Supplementary file 1 — Supplementary material. [file mmc1.docx]

| **Supplemental Table S1: Per-pack cigarette price at baseline (2016) and during a 5-year follow-up period (2018–2022) among 2,324 California households participating in the NielsenIQ Consumer Panel in every year from 2016 through 2022, California, United States.** | | | | | | | | |
| --- | --- | --- | --- | --- | --- | --- | --- | --- |
| Per-pack price (inflated to 2022 US$) | Baseline | Follow-up years | | | | | | Absolute change between 2016 and the 5-year average |
|  | 2016 | 2018 | 2019 | 2020 | 2021 | 2022 | 5-year average |  |
| All | $7.0 | $9.1 | $9.0 | $9.4 | $9.2 | $8.7 | $9.1 | $2.1 |
| Low-income | $7.0 | $8.7 | $8.7 | $8.7 | $9.0 | $8.9 | $8.8 | $1.8 |
| Middle-income | $6.7 | $9.6 | $9.8 | $9.7 | $9.1 | $9.2 | $9.5 | $2.8 |
| High-income | $7.1 | $9.2 | $8.9 | $9.5 | $9.3 | $8.6 | $9.1 | $2.0 |

Note: HH = household. Low-income: <200% FPL; Middle-income: 200% to <400% FPL; High income: ≥400% FPL.

**Supplemental Table S2: Sample size and sensitivity analysis of cigarette purchasing behaviors and financial burden of cigarette spending at baseline (2016) and in 2018 among 3,883 California households participating in the NielsenIQ Consumer Panel in both 2016 and 2018, California, United States.**

| Sample size | Baseline | Follow-up year | |  |
| --- | --- | --- | --- | --- |
|  | 2016 | 2018 | |  |
| No. of HHs | | | |  |
| All | 3883 | 3883 | |  |
| Low-income | 699 | 695 | |  |
| Middle-income | 1156 | 1079 | |  |
| High-income | 2028 | 2109 | |  |
| No. of HHs that purchased cigarettes in the respective year | | | |  |
| All | 308 | 253 | |  |
| Low-income | 70 | 82 | |  |
| Middle-income | 130 | 70 | |  |
| High-income | 108 | 101 | |  |
|  | 2016 | | 2018 | p-value of the year indicator in GEE model^*^ |
| **Cigarette purchasing behavior outcomes:**   1. % of HHs that purchased cigarettes | | | | |
| All | 10.3 | | 8.3 | **0.01** |
| Low-income | 11.5 | | 12.8 | 0.08 **^a^** |
| Middle-income | 12.8 | | 7.5 | **0.02** |
| High-income | 8.4 | | 6.1 | **<0.01 ^a^** |
| 1. No. of packs purchased by cigarette-purchasing HHs | | | | |
| All | 100.3 | | 65.3 | **0.01** |
| Low-income | 89.2 | | 61.5 | 0.39 |
| Middle-income | 120.8 | | 57.2 | 0.11 |
| High-income | 93.8 | | 73.6 | **0.03** |
| **Financial burden of cigarette spending outcomes:**   1. Annual cigarette spending (inflated to 2022 US$) among cigarette-purchasing HHs | | | | |
| All | $625 | | $566 | 0.39 |
| Low-income | $549 | | $516 | 0.28 |
| Middle-income | $721 | | $496 | **0.04** |
| High-income | $617 | | $657 | 0.94 |
| 1. % of annual HH income spent on cigarettes among cigarette-purchasing HHs | | | | |
| All | 1.7 | | 1.4 | 0.39 |
| Low-income | 3.5 | | 2.3 | 0.10 |
| Middle-income | 1.4 | | 0.9 | **0.02** |
| High-income | 0.3 | | 0.5 | 0.09 |

Note: HH = household. Low-income: <200% FPL; Middle-income: 200% to <400% FPL; High income: ≥400% FPL.

^*^ The GEE model on the outcome was specified as a function of a year indicator (=0 for 2016; =1 for 2018) as well as covariates including household composition, presence of children, type of residence, race/ethnicity, household head’s age, education, employment status, marital status, and urban/rural status

^a^ p-value =0.03 to test whether the change between 2016 and 2018 differs statistically significantly for low- vs. high-income group.
